# Suppurative thyroiditis due to aspergillosis: a case report

**DOI:** 10.1186/1752-1947-8-379

**Published:** 2014-11-21

**Authors:** Suemi Marui, Ariella Cabral de Lima Pereira, Raquel Maria de Araújo Maia, Eduardo Ferreira Borba

**Affiliations:** 1 Unidade de Tireoide-Disciplina de Endocrinologia e Metabologia, Avenida Dr Arnaldo 455 Sala 4305, 01246-903 Sao Paulo, SP, Brazil; 2 Disciplina de Reumatologia-LIM/17, Avenida Dr Arnaldo 455 Sala 3190, 01246-903 Sao Paulo, SP, Brazil

**Keywords:** *Aspergillus*, Suppurative thyroiditis, Thyrotoxicosis

## Abstract

**Introduction:**

*Aspergillus*, a nosocomial agent, is the most common fungal cause of suppurative thyroiditis. Most patients with *Aspergillus* thyroiditis have disseminated infection, primarily with lung compromise. Late diagnosis and treatment, severity of immunosuppressive state and thyroid hormone overload contribute to extremely high mortality rates.

**Case presentation:**

We describe a 20-year-old Caucasian man receiving corticosteroid suppression therapy for systemic lupus erythematosus. He presented persistent fever with neck pain and pulmonary infection. Piperacillin/tazobactam was initiated but after 2 days he developed hypoxemia, vascular shock, severe anemia, lymphopenia, and high C-reactive protein. Thyroid ultrasound revealed well-defined hypoechogenic clusters in both lobes and laboratorial thyrotoxicosis but low triiodothyronine concentration. A purulent substance was obtained on fine needle aspiration and drained. Amphotericin B and fluconazole were added but he had unfavorable evolution and died. *Aspergillus fumigatus* was defined only 2 days after his death.

**Conclusions:**

This case serves to alert clinicians to the possibility of infectious thyroiditis and reinforces the high risk of aspergillosis in immunocompromised patients. Therefore, management including voriconazole as first-line treatment or amphotericin B, in association with broad-spectrum antibiotic therapy, should be adopted to improve treatment outcome.

## Introduction

Infectious thyroiditis is relatively rare because the thyroid is remarkably resistant to infection due to its high iodine content, hydrogen peroxide production, blood supply network, abundant lymphatic drainage, and encapsulated location [1]. The main routes of infection include hematogenous or lymphatic spread and directly from adjacent infected tissue. Predisposing factors include previous thyroid disease, earlier infection at distal site, local trauma, and particularly immunocompromised conditions, such as chemotherapy and high corticosteroid therapy. Suppurative or infectious thyroiditis is painful; subacute or acute etiology must be determined to define treatment and prognosis. Local signs and symptoms of infection are indistinguishable among infectious thyroiditis. Both present with painful thyroid and thyroid enlargement, dysphagia and dysphonia. Transient thyrotoxicosis due to the release of thyroid hormone from follicular cell damage can occur. Corticosteroid therapy masks fever.

*Aspergillus*, a nosocomial agent, is the most common cause of fungal thyroiditis [2]. Most patients with fungal thyroiditis have disseminated fungal infection, primarily with lung compromise [2]. Delay in diagnosis and treatment, severity of immunosuppressive state and thyroid hormone overload contribute to extremely high mortality rates. We describe a patient on corticosteroid suppression therapy for systemic lupus erythematosus (SLE) diagnosed with *Aspergillus* thyroiditis with unfavorable evolution to alert clinicians to this rare and severe condition which is invariably overlooked.

## Case presentation

A 20-year-old Caucasian man was diagnosed with SLE due to polyarthritis, pleuritis, positive antinuclear antibodies (ANA on human epithelial type 2; HEp-2), positive anti-double-stranded DNA, and biopsy-proven class IV glomerulonephritis. Methylprednisolone 1g/day was started for 3 days followed by prednisone 1mg/kg/day. After 1 month, active renal disease persisted and methylprednisolone treatment was repeated. Thereafter, severe pancytopenia and cytomegalovirus infection were identified. Gancyclovir was administered for 21 days with good response. Cyclophosphamide was started (500mg) but after 1 month he presented daily fever and neck pain. At physical examination, an anterior painful cervical prominence was observed; heart rate 122 beats per minute, temperature 37.9°C and oxygen saturation 91%. Piperacillin/tazobactam was started as pulmonary infection was detected. After 2 days, he developed hypoxemia, vascular shock, severe anemia, lymphopenia (300/mm^3^), and high C-reactive protein (137.5mg/L; normal range, NR, <5). Thyroid ultrasound revealed well-defined hypoechogenic clusters in both lobes. Thyroid-stimulating hormone (TSH) was suppressed, free thyroxine (T_4_) >74.6pmol/L (NR 7.7 to 18), total T_4_ 189pmol/dL (NR 90 to 154), total triiodothyronine (T_3_) 1.15nmol/L (NR 1.23 to 1.85), and high serum thyroglobulin (650.6μg/L). A purulent substance was obtained on fine needle aspiration (FNA) and drained. Cytological analysis showed only pus cells. Thyrotoxicosis due to infectious thyroiditis was established. Fungal culture of thyroid abscess was partial positive. Amphotericin B and fluconazole were introduced. Tracheal and blood cultures were negative. Another thyroid ultrasound revealed persistent small clusters. He underwent drainage again but no secretion was obtained. He died at day 6 of hospital admission with suppressed TSH, lower free T_4_ but still high (47.6pmol/L) and low total T_3_ (0.60nmol/L). A culture of thyroid secretion took 10 days to reveal *Aspergillus fumigatus*, identified by septate hyphae, <5μm in thickness, with branching at acute angles, 2 days after his death.

## Discussion

Painful thyroid has two main causes: subacute thyroiditis and acute infectious thyroiditis [3]. Subacute thyroiditis is by far the most common cause of thyroid pain. Nevertheless, acute infectious thyroiditis or suppurative thyroiditis is a potentially life-threatening condition. As pain is the common presentation, clinical pictures can be used for differential diagnosis. Subacute thyroiditis frequently manifests with three classical phases: transient thyrotoxicosis, followed by transient hypothyroidism and usually euthyroidism. Patients report previous viral infection with low fever and malaise. Therapy aims to relieve pain; evolution is favorable and self-limited. Subacute thyroiditis rarely causes severe thyrotoxicosis [1].

Conversely, infectious acute thyroiditis is a potentially life-threatening disorder whose prognosis depends on prompt recognition and treatment [4]. Bacterial, fungal, and even parasitic organisms have been documented as etiologic agents. Phlogistic signs are often seen. Abscess drainage and antimicrobial therapy, including antifungal, must be performed. In contrast to subacute thyroiditis, thyroid dysfunction is rarely noted in this condition.

Of interest, our case showed all the classical signs and symptoms of subacute thyroiditis and had a previous cytomegalovirus infection. However, he was already on corticosteroid therapy which is effective to treat subacute thyroiditis. Immunocompromised status that occurs in patients with human immunodeficiency virus, corticosteroid therapy, organ transplant and chemotherapy, is prone to atypical infections, rare etiologies, bizarre presentations and unusual primary infectious foci.

Common pathogens are encountered with greater frequency in a dose-dependent manner during therapy with glucocorticoids. Most patients present viral infections, herpes or cytomegalovirus, as our patient, but may also have *Staphylococcus*, candidiasis and *Strongyloides. Aspergillus* is increasingly recognized as an important nosocomial pathogen in immunocompromised patients but it is difficult to diagnose antemortem and typically has a fatal outcome. At autopsy, thyroid commitment is the result of a disseminated infection even in the absence of thyroid dysfunction [5]. Antemortem diagnosis is reached by direct microscopy and culture after FNA or by biopsy in most reported cases. Thyroid FNA is the easiest and fastest method of diagnosis, and cytological analysis reveals profuse purulent infiltration with hypha. In this regard, aerobic and anaerobic cultures of FNA specimens must also be performed, but fungal cultures are also mandatory. Unfortunately, fungal culture involves a lengthy process. Therefore, our management included broad-spectrum antibiotics and metronidazole. Amphotericin B and fluconazole however, were only added after positive fungal culture and, albeit unsuccessful, abscess drainage. Thyroidectomy should be performed only to debride necrotic tissue and to remove infected tissue if abscess persists. Surgery has more death risk as it requires a patient in better condition to tolerate surgery, as bleeding and hemodynamic instability are common.

Our service does not provide voriconazole, the best treatment to *Aspergillus* infection [6]. Some authors suggest that fluconazole should be added to treatment for immunocompromised infected patients, as well as *Aspergillus* serology [2]. As the primary site is frequently located in the upper respiratory tract, nasal or orotracheal samples must be collected for repetitive fungal culture, especially with mild lung infiltration. Growth of *Aspergillus* in blood cultures is rare but highly specific [7]. It is noteworthy that fungal cultures from orotracheal secretion and blood were negative in our patient. In our patient, fungal cultures took 10 days to reveal *Aspergillus* in colonies.

All but one anecdotal case of *Aspergillus* thyroiditis reported previously had unfavorable outcomes, which suggest delays in diagnosis, representing a marker of poor prognosis [2,8-11].

Also of interest, our patient presented low T_3_ concomitant with high T_4_ level. After follicular thyroid damage, T_4_ is disproportionately elevated relative to T_3_, reflecting intrathyroidal T_4_/T_3_ ratio. Conversely, low T_3_ observed in our patient could also reflect worse prognosis, as identified in euthyroid sick syndrome [12].

## Conclusions

This case alerts clinicians to the possibility of infectious thyroiditis and reinforces the high risk of aspergillosis in immunocompromised patients. Therefore, management including voriconazole and amphotericin B, in association with broad-spectrum antibiotic therapy, should be adopted to improve treatment outcome.

## Consent

Written informed consent was obtained from the mother of the patient for publication of this case report. A copy of the written consent is available for review by the Editor-in-Chief of this journal.

## Abbreviations

FNA: Fine needle aspiration; NR: Normal range; SLE: Systemic lupus erythematosus; T_3_: Triiodothyronine; T_4_: Thyroxine; TSH: Thyroid-stimulating hormone.

## Competing interests

The authors declare that they have no competing interests.

## Authors’ contributions

SM attended, interpreted the patient data, wrote and submitted the manuscript. ACLP and RMAM attended the patient at our Intensive Care Unit. EFB performed diagnosis and treatment of systemic lupus erythematosus. All authors read and approved the final manuscript.

## References

[B1] Pearce EN Farwell AP Braverman LE Thyroiditis N Engl J Med 2003 348 2646 2655 10.1056/NEJMra021194 12826640

[B2] Goldani LZ Zavascki AP Maia AL Fungal thyroiditis: an overview Mycopathologia 2006 161 129 139 10.1007/s11046-005-0239-3 16482384

[B3] Farwell AP Braverman LE, Cooper DS Sporadic Painless, Painful Subacute and Acute Infectious Thyroiditis Werner and Ingbar’s The Thyroid: A Fundamental and Clinical Text 2013 10 Philadelphia: Lippincott Williams & Wilkins 414 429

[B4] Paes JE Burman KD Cohen J Franklyn J McHenry CR Shoham S Kloos RT Acute bacterial suppurative thyroiditis: a clinical review and expert opinion Thyroid 2010 20 247 255 10.1089/thy.2008.0146 20144025

[B5] Hori A Kami M Kishi Y Machida U Matsumura T Kashima T Clinical significance of extra-pulmonary involvement of invasive aspergillosis: a retrospective autopsy-based study of 107 patients J Hosp Infect 2002 50 175 182 10.1053/jhin.2001.1170 11886192

[B6] Limper AH Knox KS Sarosi GA Ampel NM Bennett JE Catanzaro A Davies SF Dismukes WE Hage CA Marr KA Mody CH Perfect JR Stevens DA and on behalf of the American Thoracic Society Fungal Working Group An official American Thoracic Society statement: treatment of fungal infections in adult pulmonary and critical care patients Am J Respir Crit Care Med 2011 183 96 128 10.1164/rccm.2008-740ST 21193785

[B7] Denning DW Therapeutic outcome in invasive aspergillosis Clin Infect Dis 1996 23 608 615 10.1093/clinids/23.3.608 8879787

[B8] Ayala AR Basaria S Roberts KE Cooper DS *Aspergillus* thyroiditis Postgrad Med J 2001 77 336 10.1136/pmj.77.907.336 11320281 PMC1742019

[B9] Hornef MW Schopohl J Zietz C Hallfeldt KKJ Roggenkamp A Gartner R Heesemann J Thyrotoxicosis induced by thyroid involvement of disseminated *Aspergillus fumigatus* infection J Clin Microbiol 2000 38 886 887 10655408 10.1128/jcm.38.2.886-887.2000 PMC86235

[B10] Vogeser M Wanders A Haas A Ruckdeschel G A four-year review of fatal Aspergillosis Eur J Clin Microbiol Infect Dis 1999 18 42 45 10.1007/s100960050224 10192713

[B11] Thada ND Prasad SC Alva B Pokharel M Prasad KC A rare case of suppurative aspergillosis of the thyroid Case Rep Otolaryngol 2013 2013 4 10.1155/2013/956236 PMC3789295 24159398

[B12] Mebis L Debaveye Y Visser TJ Van den Berghe G Changes within the thyroid axis during the course of critical illness Endocrinol Metab Clin North Am 2006 35 807 821 10.1016/j.ecl.2006.09.009 17127148

